# Trusted Multi-Domain DDoS Detection Based on Federated Learning

**DOI:** 10.3390/s22207753

**Published:** 2022-10-12

**Authors:** Ziwei Yin, Kun Li, Hongjun Bi

**Affiliations:** School of Electronic and Information Engineering, Beijing Jiaotong University, Beijing 100044, China

**Keywords:** DDoS, federated learning, reputation evaluation, knowledge base

## Abstract

Aiming at the problems of single detection target of existing distributed denial of service (DDoS) attacks, incomplete detection datasets and privacy caused by shared datasets, we propose a trusted multi-domain DDoS detection method based on federated learning. Firstly, we divide the types of DDoS attacks into different sub-attacks, design the federated learning dataset for DDoS detection in each domain, and use them to realize a more comprehensive detection method of DDoS attacks on the premise of protecting the data privacy of each domain. Secondly, in order to improve the robustness of federated learning and alleviate poisoning attack, we propose a reputation evaluation method based on blockchain, which estimates interaction reputation, data reputation and resource reputation of each participant comprehensively, so as to obtain the trusted federated learning participants and identify the malicious participants. In addition, we also propose a combination scheme of multi-domain detection and distributed knowledge base and design a feature graph of malicious behavior based on a knowledge graph to realize the memory of multi-domain feature knowledge. The experimental results show that the accuracy of most categories of the multi-domain DDoS detection method can reach more than 95% with the protection of datasets, and the reputation evaluation method proposed in this paper has a higher ability to identify malicious participants against the data poisoning attack when the threshold is set to 0.6.

## 1. Introduction

With the rapid development of 5G, network security problems are becoming increasingly serious, and a DDoS (distributed denial of service) attack is destructive, particularly [1,2,3]. DDoS attacks can be divided into different sub-attacks according to different perspectives [4], such as network layer DDoS attacks [5], application layer DDoS attacks [6], DRDoS (reflective DDoS) attacks [7], LDDoS (low-rate DDoS) attacks [8] and botnet DDoS attacks [9]. Due to the wide variety of DDoS attacks, the types of attack dataset collected by a single user is often not comprehensive, and the detection model trained based on this dataset has certain limitations. If datasets transfer from all domains to one party for training, it will demand the performance of the central server, and large-scale data can also cause high latency when uploading. At the same time, some organizations sometimes do not want to make all the flow information in their network domain public, and if this sensitive data is transmitted, it may be leaked or misused in the process, resulting in privacy and security issues. With protection of users’ privacy making dataset sharing a limiting factor, how to use the dataset collected from multiple domains without compromising data privacy, so as to detect DDoS attack flow comprehensively, is an urgent problem to be solved.

The proposal of FL (federated learning) [10] solves the problem of data privacy effectively. FL aims to build a global model by enabling multi-participant with local data to train the same model in a distributed manner, and only by exchanging model parameters or intermediate results without exchanging sample data. However, it brings other hidden risks of privacy and security [11], such as inference attack and poisoning attack. Inference attack refers to that intermediate parameters like gradient and loss of the model are exposed to the outside directly without encryption and intercepted or reasoned maliciously, but there are good solutions for such attacks currently [12]. Poisoning attack is the mainstream of current research. It mainly refers to malicious behaviors initiated by semi-honest or malicious nodes in participants, including providing poisoning models or training poisoning data to improve activity and contribution [13]. For poisoning attack, some scholars put forward the method of using a reputation evaluation mechanism, but this kind of method needs interaction with the training model of each round. It will lead to vulnerability to malicious reasoning models during evaluation and increase privacy and security risks. In addition, too often interacting with blockchain would lead to consuming a large number of communication resources.

Therefore, in this article, we present a multi-domain DDoS attack detection method based on trusted federated learning for data privacy protection. Firstly, use blockchain to register and set the initial reputation of FL participants, and wait for processing task applications. Secondly, preprocess the local FL dataset, respectively, after the task application is approved, and use ML (machine learning) or DL (deep learning) methods to train the preprocessed local dataset to obtain the local model. Then, the local model parameters will be encrypted and uploaded to the aggregation server to generate the global model, and the final global model is generated by iterative training. Finally, upload the relevant information of the task to the blockchain for audit, use the smart contract to calculate the comprehensive reputation and update the reputation of participants.

The contributions of this article are as follows:

(1) A multi-domain DDoS detection method based on FL is proposed. The types of DDoS attacks are divided into five categories: network layer DDoS, application layer DDoS, LDDoS, botnet DDoS and DRDoS. Based on the classification, the labels of FL datasets in each domain are redesigned to realize multi-domain DDoS detection.
(2) A reputation evaluation method of Federated participants based on blockchain is proposed. The reputation of FL participants is evaluated and calculated from three perspectives: interaction reputation, data reputation and resource reputation, which realizes the effective evaluation of reputation on the basis of reducing the number of interactions.
(3) Build a topology network for multi-domain DDoS attack and detection and carry out multi-domain DDoS detection under the above trusted Federation learning. The proposed scheme can distinguish malicious participants from trusted participants effectively after simulating the FL data poisoning attack.
(4) A knowledge export scheme of FL is designed, which stores the feature knowledge after multi-domain learning to the feature graph of malicious behavior, and stores the model to the model base, so as to realize the construction and update of the distributed knowledge base from local domain knowledge to multi-domain knowledge under the condition of privacy protection.

This study includes five sections. The Section 1 introduces to trusted multi-domain DDoS detection based on federated learning. The Section 2 deals with the literature review of studies related to federated learning DDoS detection and reputation evaluation of participant. The Section 3 gives detailed information about the methodology of the study. The results obtained from the method mentioned in the methodology section are discussed in the Section 4. In the Section 5, conclusion about the study has been provided.

## 2. Related Works

In this section, we review the previous work of the development of DDoS attack detection methods, DDoS detection based on FL, and reputation evaluation methods of FL participants.

### 2.1. DDoS Attack Detection Method

At present, the existing detection methods for DDoS attacks are mainly based on ML or DL. Build an intrusion detection classifier by selecting an appropriate ML or DL model [14,15,16,17] and analyze the difference in features between normal flow data and abnormal flow of the network, so as to judge the type of attack. Jing et al. [18] investigated the existing DDoS-related data and detection methods of DDoS and worm attacks, divided the datasets into packet level datasets and flow level datasets, and introduced the detection technologies from application layer DDoS, network layer DDoS, LDDoS and botnet DDoS. From the perspective of detection methods, these methods can be divided into supervised ML or unsupervised ML [19], offline detection or real-time online detection [20]. In addition, the existing DDoS detection methods [5,6,7,8,21,22] only aim at a specific category of attack, such as application layer DDoS attack, and lack a comprehensive method to detect different categories of DDoS as a whole. In a word, the above methods usually require a large number of training sets, especially for the purpose of achieving a variety of attack categories, so the ML model trained in a single domain is difficult to achieve high detection ability.

### 2.2. DDoS Detection Based on FL

The DDoS detection methods based on FL solve the problem of insufficient data volume effectively on the premise of ensuring the privacy of the data owner. It uses the datasets mastered by each participant to train the local model and share the model parameters to realize multi-party joint modeling, which can be completed without uploading data. Some scholars apply FL technology to intrusion detection [23,24], and construct distributed network intrusion detection systems. It is proved that intrusion detection based on FL can improve the efficiency and confidentiality of training and achieve an accuracy similar to centralized ML after data sharing. Since datasets of FL are located on different clients, data samples and labels may follow different distributions. This non-iid (non-independent identically distribution) may significantly reduce the performance of FL [25]. Therefore, Zhao et al. [26] apply the long-term and short-term memory model to the FL framework, and Zhang et al. [27] propose a hierarchical aggregation algorithm based on K-means, which improves the impact of datasets on DDoS detection capabilities. However, most literature only detect a specific category of DDoS attacks [28], there are few schemes to realize multi categories DDoS detection under multi-domain.

### 2.3. Reputation Evaluation Method of FL Participants

From the perspective of the security of FL, poisoning attack is the most prominent security problem. At present, there are two patterns to mitigate poisoning attacks, one is a fault-tolerant global model aggregation algorithm [29], and the other is to identify malicious participants by designing an evaluation mechanism for local models [30,31]. For example, Xu et al. [32] use the reputation evaluation scheme of cosine similarity between the model gradient and the global model gradient. Recently, some scholars have proposed to combine the reputation evaluation mechanism of blockchain. Park et al. [33] propose a miner selection method based on reputation reliability, which realizes reliable joint learning by using a subjective logic model with multiple weights and uses blockchain for reputation management. Zhang et al. [34] introduced model cross entropy to calculate reputation value through model quality parameters to evaluate workers’ reliability. Uprety et al. [35] estimate binomial Bayesian reputation score based on beta distribution, and the posterior reputation score updated each time is the combination of a priori reputation score and new feedback. In addition, in order to make FL more equitable, some scholars have proposed a blockchain-based audit mechanism to record and reward high-quality participants. Mugunthan et al. [36] propose an audit mechanism, which uses Ethereum and smart contracts to encourage good behavior and rewards all parties with cryptocurrencies according to the quality of their contributions. The methods for mitigating poisoning attacks based on blockchain mentioned above are effective to a large extent, but most need to evaluate each round of models. Once the model parameters are exposed, the risk of being maliciously reasoned will increase greatly. At the same time, these methods will also increase the burden of communication.

We organize the above research into Table 1, where N means No and Y means Yes.

In order to solve the problems of existing DDoS attack detection based on ML and FL, such as single detection target, incomplete detection dataset, and privacy caused by shared dataset, we propose a multi-domain DDoS detection method based on FL. In our method, DDoS types are divided into five categories, so as to realize a more comprehensive detection method for multi-domain and multi type DDoS attacks. In addition, in order to improve the robustness of our method and mitigate poisoning attacks, we propose a reputation evaluation method based on blockchain to obtain trusted FL participants. In addition, we also propose a combination scheme of multi-domain detection and distributed knowledge base and design a feature graph of malicious behavior based on knowledge graph to realize the memory of multi-domain feature knowledge. Finally, based on the experimental topology, we verify the scheme of our proposals.

## 3. Multi-Domain DDoS Detection Method Based on Trusted FL

In this section, we introduce the overall framework of multi-domain DDoS detection based on trusted FL firstly. Then, the implementation scheme and corresponding key technologies of the three main modules in the framework are introduced in detail. Finally, we describe the calling relationship between the three modules and the overall implementation process.

### 3.1. Framework Overview

In order to achieve a more comprehensive detection of DDoS attack targets, we simulate a variety of DDoS attacks and normal flow, classify the simulated DDoS attacks, redesign the datasets, and complete the FL DDoS detection by using the designed multi-domain datasets. At the same time, in order to prevent poisoning attacks caused by the introduction of FL, we add a reputation evaluation scheme based on blockchain to select trusted participants to complete trusted federated DDoS detection. Finally, we store the FL models and effective features in the knowledge base and deploy models in the gateway to complete online detection guided by the knowledge base, so that even if there is no specific attack type in this domain, it can be divided into its own attack category under the knowledge of other domains.

**Figure 1:**
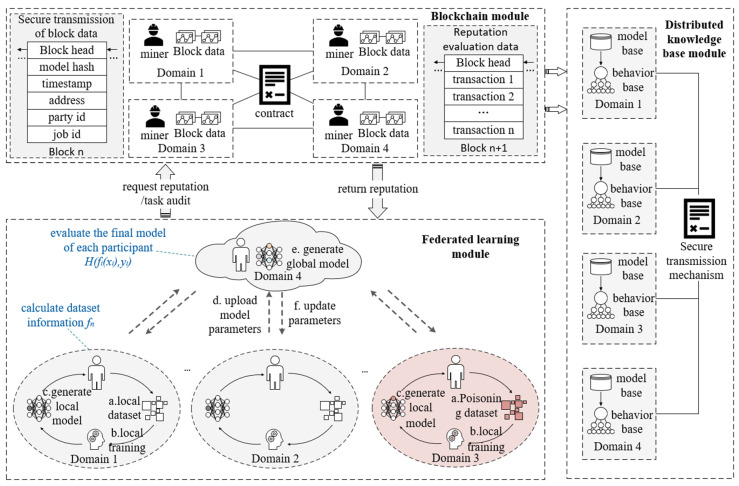
Overall framework.

In this paper, we summarize the scheme as three modules: federated learning module, blockchain module and distributed knowledge base module. Each module is deployed in multiple domains, with the architecture shown in Figure 1.

The main contents of the federated learning module are as follows. Firstly, the local model is generated through the steps of collecting local datasets and starting local training. Then, the model parameters of all domains are uploaded to generate a global model. Finally, the model parameters of each domain are updated according to the generated global model. This module enables each domain to learn global DDoS knowledge from datasets owned by all domains, so as to obtain stronger DDoS detection capabilities. For example, the dataset collected by domain 1 does not include HTTP-GET attacks in application layer DDoS, but HTTP-GET attacks have already occurred in domain 2. The scheme proposed in our paper will enable domain 1 to learn the knowledge of domain 2 through FL, so as to ensure that if HTTP-GET attacks occur in network domain 1 later, they can also be classified as application layer attacks correctly. However, it should be noted that we consider designing untrusted participants to improve the robustness of our scheme, as shown in domain 3 in Figure 1. We consider the data poisoning attack, especially the flip label attack, in which the label of the training example was flipped to the target class. For example, in MNIST a “1–7” flipping refers to training on the image of “1” but using “7” as a label.

The blockchain module is deployed and maintained by all domains participating in FL and implements the reputation evaluation mechanism and the secure transmission mechanism. The reputation evaluation mechanism not only processes the reputation request of the federated learning module and feeds back the reputation value, but also audits the tasks of the federated learning module to complete the updating and maintenance of the reputation value. The secure transmission mechanism ensures that the model cannot be tampered with in the interaction process.

The distributed knowledge base module realizes the distributed construction of the local knowledge base based on the blockchain. In this module, the FL model and the extracted effective feature are stored in the model base and the behavior base, respectively, through the secure transmission mechanism of the blockchain module, so as to store and update the knowledge and finally provide knowledge feedback for malicious behavior detection.

### 3.2. Federated Learning Module

**Figure 2:**
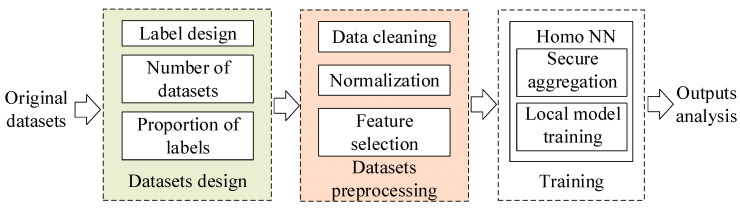
Training flow chart of federated learning module.

The federated learning module includes DDoS datasets design of each domain, data preprocessing and offline training, which is shown in Figure 2.

#### 3.2.1. Dataset Design

Considering that there are fewer datasets for comprehensive detection of DDoS at present, the datasets are designed in the federated learning module first.

We use tools and scripts to simulate attacks and normal flow [5], shown as Table 2. For example, SlowHTTPTest is used to simulate slow headers, slow body, and slow headers belong to LDDoS attacks by setting the content length of the application layer to be large or the sending rate parameter to be lower than the expected rate, so as to continuously occupy HTTP connection sessions. Python’s scapy library is used to forge requests to some UDP protocol servers, such as TFTP servers, which can conduct IP spoofing without verifying the identity of the sender to form a more harmful attack. Then, to collect the flow we simulated at the network entrance of each domain for a certain period of time to get the original data flow. Through CICFlowMeter (https://github.com/CanadianInstituteForCybersecurity/CICFlowMeter/ (accessed on 7 October 2022)), a tool to extract flow feature, the original feature information of DDoS attacks and normal flow in each domain is obtained. There are 22 types of simulated attacks, which are described as ACK, UDP, SYN, SlowBody, Shrew, SlowHeaders, SlowRead, Ares, BYOB, Miral, Zeus, IRC-Botnet, TFTP, Memcached, DRDoS_SSDP, DRDoS_NTP, Chargen, DRDoS_SNMP, CC, HTTP-Get, HTTP-Flood and HTTP-Post.

Since the network flow collected by each domain may not include all the above attack types, we design datasets suitable for multi-domain DDoS detection, and divided the above 22 attack types into five categories, namely botnet DDoS attack, application layer DDoS attack, slow DDoS attack, DRDoS attack and network layer DDoS attack, which is also in Table 2. The label design method we proposed improves the label missing situation when there are many attack types in training datasets, so it can mitigate the influence of non-iid to a certain extent.

The datasets generated by CICFlowMeter in each domain have the same feature space, including 84 kinds of feature information, such as ID, quintuple information (source IP address, destination IP address, source port, destination port and protocol), stream features and packet features. Considering that the feature relationship of datasets is similar to horizontal FL, the DDoS detection scheme designed in this paper is a horizontal FL method.

In addition to the above classification, we add each to flow a feature of ID information of each dataset in their respective domains. In practice, the send time of each data flow is different, so each data flow should be given a new ID. In addition, if the local dataset still has non-iid conditions after label design, we improve learning ability by sharing a small part of the data in consideration of the number of datasets and the proportion of labels.

#### 3.2.2. Data Preprocessing

The outputs of the dataset design step still have abnormal data, non-standard data and redundant features, so they should be preprocessed.

The data preprocessing proposed in this paper includes three major steps: data cleaning, data normalization and feature selection. Firstly, deal with the outliers in the dataset, delete the row where the missing value is located or set the missing value as a fixed value, and use coding to convert qualitative features into quantitative features. Secondly, min-max standardization is used to normalize the data and convert the data to the range of 0 and 1, so as to convert different features into the same dimension and improve the convergence speed of the model. Finally, select the features, remove the irrelevant features such as IP address and timestamp, and use the random forest as the selection method to sort the importance of the features. Integrate the factors of training time and training effect, and finally, we select the first 53 features.

#### 3.2.3. Offline Training

The offline training part is mainly responsible for generating the FL model with a high detection rate under the premise of protecting privacy. The specific algorithm is described as Algorithm 1. The algorithm used in this paper is HomoCNN, jointly implemented by the aggregator and the participants to realize the local CNN training and secure aggregation process.
**Algorithm 1** HomoCNNb (Horizontal Convolution Neural Network) Algorithm**Input:** Training dataset and test dataset of *K* participants; iteration *N*; local epoch *E* 1: /*Central server*/ 2: Initialize model parameters $M^{\left(0\right)}$; 3: **for**
*n* = 0; *n* < *N*; *n*++ **do**: 4:        **for**
*k* = 0; *k* < *K*; *k*++ **do**: 5:               $C_{k}^{\left(n\right)}\leftarrow$ the encrypted model parameters of participant *k*; 6:        **end for** 7:         $M^{\left(n+1\right)}\leftarrow \sum C_{k}^{\left(n\right)}/K\;$; 8: **end for** 9: /*Client update*/ 10: **for**
*k* = 0; *k* < *K*; *k*++ **do**: 11:          $M_{k}^{\left(n-1\right)}$ 
← the global CNN model parameters; 12:          **for** e = 0; e < *E*, e++ **do**: 13:                  $M_{k}^{\left(n-1\right)}\leftarrow M_{k}^{\left(n-1\right)}-\eta \nabla l\left(M_{k}^{\left(n-1\right)}\right)$, where $l\left(M_{k}^{\left(n-1\right)}\right)$ is loss function; 14:          **end for** 15:          update local CNN model $M_{k}^{\left(n\right)}\leftarrow M_{k}^{\left(n-1\right)}\;$; 16:          encrypt local CNN model $C_{k}^{\left(n\right)}\leftarrow M_{k}^{\left(n\right)}+\sum_{i\neq j}R_{i,j}^{\left(n\right)}\;$$,\;\mathrm{where}\;R_{i,j}$ is random mask; 17: **end for**

**Figure 3:**
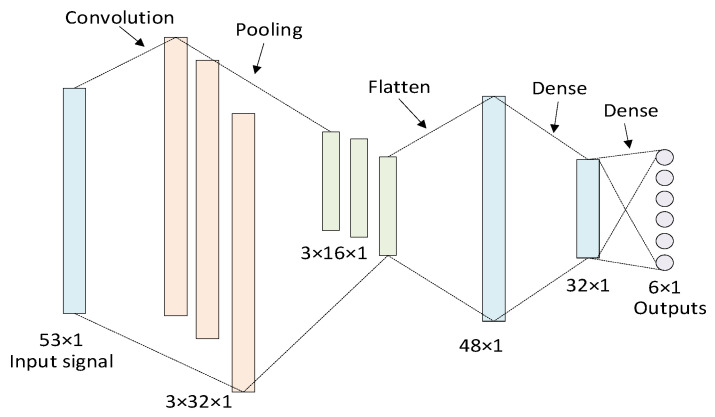
Training model of local convolutional neural network.

The local training model in the offline training is realized by CNN, including 1 input layer, 1 convolution layer, 1 pooling layer and 2 dense layers, and the specific network is shown in Figure 3. The input layer receives data after preprocessing with a dimension of 53 × 1. The convolution layer extracts the input features and their relationships, and generates three feature maps using three 2 × 2 convolution kernels. The pooling layer uses a 2 × 2 filter to pool the three feature maps and reduce its dimension. Flatten layer is used for dimension transformation to realize the flattening of dimensions. The dense layers realize the mapping between the relations of features, in the meantime, the last dense layer connects the softmax layer to realize the output of probability.

The locally trained models will achieve privacy protection of datasets and model parameters through security aggregation. On the one hand, the CNN models are trained independently by each participant to protect the dataset of each participant. In Algorithm 1, the interaction of each participant only needs to upload its model parameters *C_k_*^(*i*)^ to achieve average aggregation, where the weight of each model is 1/*K* in aggregation. On the other hand, the security aggregation of the model is realized by using a single mask. Specifically, model parameters encrypted by random mask are translated to central server, then aggregated by central server to obtain the decrypted global model. The key used to encrypt parameters is random mask *R_i,j_*, which is agreed between participants *i* and *j* according to the total order of participants. When encrypting model parameters, each participant will use the *R_i,j_* negotiated by them together to mask its model parameters *M_k_*^(*i*)^. If *i* < *j*, add *R_i*,*j_* to *M_k_*^(*i*)^, otherwise, subtract.

When the central server aggregates the encryption model, the encrypted results are added, and all encryption keys will be offset, but the actual input of each participant will not be exposed, so as to realize decryption. In this process, the privacy protection of model parameters is achieved.

### 3.3. Blockchain Module

The blockchain module includes the reputation evaluation mechanism and the transmission mechanism. The following will describe the two main contents of the reputation evaluation mechanism first, one is participant identity registration, information management and feedback, and the other is reputation evaluation of the participants. Then, elaborate on the transmission mechanism.

#### 3.3.1. Identity Registration, Information Management and Feedback

Identity registration is the premise for participants to participate in FL training. When registering, it is necessary to set up the only identity information party ID of FL and name description information of this participant. The blockchain calls the smart contract to register its identity, writes the information of the participants who apply and who have to participate in the FL training task into the blockchain, and waits for the task request after registration.

After registration, the blockchain can carry out information management and feedback on the participants. Among them, information management will manage the identity attributes, data attributes and job attributes of the participants, respectively, as shown in Table 3.

Identity information management is responsible to modify the online status, domain name and other attributes of participants and log out the identity after their identity registration is completed.

Data information management is responsible for adding local dataset descriptions and modifying or deleting the added dataset information. The attributes include the Ethereum address of the dataset owner, the party ID of the data owner, the data description namespace and table name, information entropy and quantity of data.

Job information management is responsible for auditing each FL training task. After completing the training task and evaluating the received models trained by other participants, the guest of the job is required to upload the evaluation score and the corresponding job ID, party ID and other information to the blockchain to facilitate the retrieval of task and task related information. At the same time, it facilitates to modification of the job ID, party ID and the cross entropy of all models of the audited task.

Information feedback provides output interface functions for attributes information mentioned above.

#### 3.3.2. Reputation Evaluation Method

**Figure 4:**
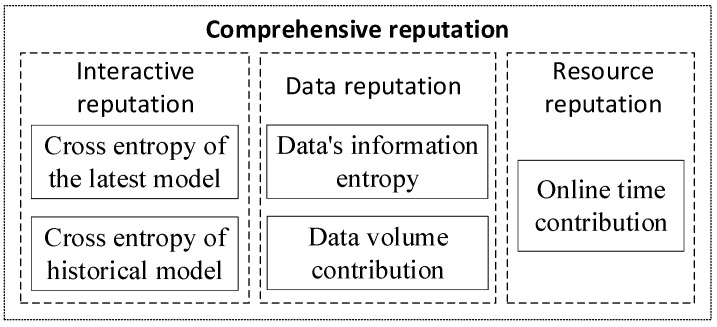
Reputation evaluation method.

The evaluation indicators of reputation include the interactive reputation of participants, data reputation and resource reputation jointly, as shown in Figure 4. Specifically, interactive reputation is influenced by cross entropy of the latest model and historical model. Data reputation is composed of the volume and information entropy of the participator’s datasets. Resource reputation is determined by the proportion of online time of the participator.

We concretize the above indicators and calculate the latest interactive reputation score *S_LATEST_* and historical interactive reputation score *S_HISTORY_*, respectively, according to the cross entropy of the model. The calculation formula is shown in (1) and (2), where *b*_1_ and *k*_1_ is the constant that maps the cross entropy *H*(*f_i_*(*x_i_*),*y_i_*) to score.

(1) $$S_{LATEST}=b_{1}-k_{1}\times H_{LATEST}\left(f_{i}\left(x_{i}\right),y_{i}\right)$$

(2) $$S_{HISTORY}=b_{1}-\frac{k_{1}}{n-1}\sum_{j=1}^{n-1}H_{j}\left(f_{i}\left(x_{i}\right),y_{i}\right)$$

Since the model transmission of the interaction process is under encryption, the aggregator cannot evaluate all the models during this process. At the same time, in order to ensure that the beginning training models that are easier to reason are not intercepted maliciously in the aggregation, the interactive reputation evaluation method we proposed only evaluates the model of the last iteration, which not only retains the original model encryption transmission process but also increases the cost of the malicious attacker’s reasoning source data. We need to calculate the cross entropy *H*(*f_i_*(*x_i_*),*y_i_*)of each model and calculate the corresponding cross entropy score *S_LATEST_* and *S_HISTORY_*. The calculation method of cross entropy of the model is shown in Formula (3), Where (*x_i_*, *y_i_*) is the dataset used to evaluate the model, *f_i_*(*x_i_*) is the final model trained by each participant, and *N* is the number of evaluation dataset.

(3) $$H\left(f_{i}\left(x_{i}\right),y_{i}\right)=-\frac{1}{N}\underset{x_{i}}{\sum }y_{i}\log\left(f_{i}\left(x_{i}\right)\right)$$

Therefore, the calculation method of interactive reputation is shown in Formula (4). In our paper, the *RVi* of the untrusted participants will be lower significantly because the poisoning dataset makes their models of low quality and hence their cross entropy score low.

(4) $$RVi=\frac{1}{2}S_{LATEST}+\frac{1}{2}S_{HISTORY}$$

Data reputation is weighted by the volume score *S_Nd_* and the data entropy score *S_Hd_* of datasets that all users hold. In order to encourage participants to provide more datasets for FL training, the score we set for the submitted dataset includes upper and lower limits, and the corresponding scores can be obtained even if the amount of dataset provided is small. Specifically, we use piecewise function *S_Nd_* to score the amount of data, and the calculation method is shown in Formula (5).

(5) $$S_{N_{d}}=\{\begin{array}{c} 40,\;\;\;N<1000\\ b_{2}+k_{2}\times N,\;1000<N<100000\\ 100,\;N>100000 \end{array}$$

Data information entropy score *S_Hd_* is expressed by normalized information entropy, and it can map the information entropy to a fixed range, shown as Formula (6), where *j* is the type of label *y_i_*.

(6) $$S_{H_{d}}=\frac{-\sum_{y_{i}}p\left(y_{i}\right)\log p\left(y_{i}\right)}{-\log\left(1/j\right)}$$

Data reputation is represented by Formula (7), which can detect the data with low quality effectively and give these data a relatively small score, especially for poisoning datasets whose quality is generally poor.

(7) $$RVd=\frac{1}{2}S_{N_{d}}+\frac{1}{2}S_{H_{d}}$$

The resource reputation is determined by the proportion of the user’s online time *St_i_*. The calculation method is shown in Formula (8), where *T_online_* is the online time of the device, and *T* is the time interval from the registration of the participant to current.

(8) $$RVr=St_{i}=\frac{T_{online}}{T}$$

To sum up, the calculation of comprehensive reputation update is shown in Formula (9), where *a*_1_, *a*_2_ and *a*_3_ are the weights corresponding to the evaluation index, and *a*_1_ *+ a*_2_ *+ a*_3_ *=* 1. The scheme of a comprehensive evaluation of interaction reputation, data reputation and resource reputation proposed has significant advantages for data poisoning attacks.

(9) $$R=a_{1}\times RVi+a_{2}\times RVd+a_{3}\times RVr$$

The reputation evaluation mechanism will calculate and evaluate the comprehensive reputation value of each FL participant, so as to feed back the evaluation results to the guest of task, and it will judge whether the comprehensive reputation of each participant is at the reputation threshold β, so as to decide whether to determine the node as the participant of FL training.

#### 3.3.3. Secure Transmission

After completing the training task, the guest of the job needs to receive the final model trained by other participants and evaluate them together to upload information such as interaction scores to the blockchain for audit. During the point-to-point transmission of the model, if the process takes too long, the data transmission time may be manipulated. Therefore, we add a secure transmission mechanism to the blockchain module to improve the security and immutability of transmission.

Store the hash value of model knowledge and feature knowledge into the blockchain. If the hash value on the blockchain is the same as the hash value of the model received through the point-to-point transmission protocol, it can be proved that the model has not been maliciously tampered with in the transmission process, and the transmission process is secure.

### 3.4. Distributed Knowledge Base Module

The distributed knowledge base module models the same category of DDoS attacks from the perspective of flow features, obtains the significant difference between the features of this category of DDoS attack and normal flow, and models different categories of DDoS detection methods to provide knowledgeable guidance for detection, so as to detect and mitigate DDoS attacks effectively.

**Figure 5:**
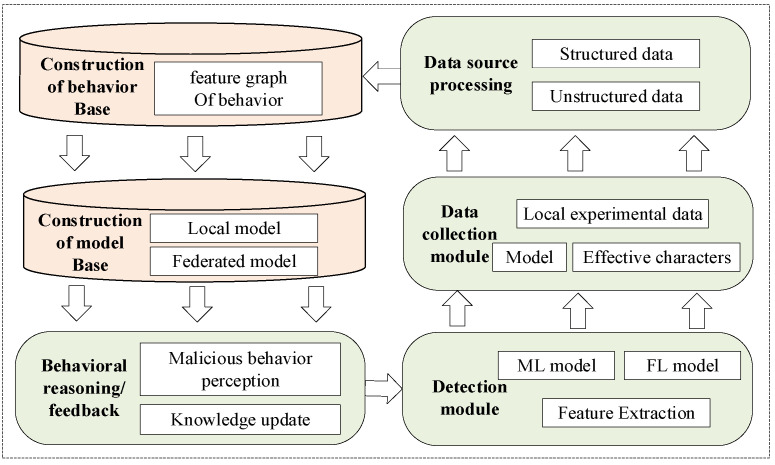
Construction of knowledge base.

The construction process of the distributed knowledge base proposed in this paper is shown in Figure 5. In the figure, the data collection module is responsible for collecting the experimental data, the models and the effective features. After processing by the data source processing module, they are classified into structured data and unstructured data. Among them, the effective features and other data are used to build the behavior base, which is responsible for remembering the malicious attack behaviors, mainly DDoS. The model data is used to construct model base to store local detection models and global detection models built by multi-domain individually or cooperatively. The knowledge base is composed of behavior base and model base. The constructed knowledge base is used for behavior reasoning and feedback, providing model information and feature knowledge for the detection module. Finally, the predicted results as experimental data of the detection module will be feedback to the data collection module, and the whole process forms closed-loop management.

Model base is mainly built based on the trained model, which includes local model and federated model. The local model is trained according to the local dataset, which has not been trained jointly, so it can only detect the attacks that have appeared locally. The detection ability of local model is limited by the attacks that does not appear in the local domain. However, the federated model is the result of the collaborative multi-domain training, which reflects the knowledge of DDoS flow received by all domains and has a more comprehensive guidance ability. The models in the model base mainly include model structure and model weight, as shown in the Formula (10).

(10) M=<M_S,M_W>

Behavior base is constructed in the form of triples based on the knowledge graph, as shown in the Formula (11).

(11) KG=<E,R,P>

where *E* = {e_1_, e_2_, …, e_i_} is the set of entities, representing objects stored in the knowledge graph. *R* = {r_1_, r_2_, …, r_j_} is the set of relationships, representing association relationships between these entities. *P* = {p_1_, p_2_, …, p_n_} is a set of attributes and a specific representation of the stored data of the knowledge graph, and any relationship and entity may have different attributes.

#### 3.4.1. Model Base

The models in the model base are mainly stored in the form of a combination of model structure and model parameters. Specifically, we take the final model generated by the participant who performs best in FL as the global model and provides a coarse-grained DDoS classification method for the detection module.

In order to identify 22 specific attack types more accurately, we train five local fine-grained models in each domain such as network layer DDoS detection model after the coarse-grained category detection is completed, which are trained by four domains, respectively, and distributed stored in the distributed knowledge base.

#### 3.4.2. Behavior Base

In the behavior base, we build a feature graph of malicious behavior. The main function of the malicious behavior feature graph is to count the traffic features corresponding to 5 categories of sub-attacks and 22 specific attack types. These corresponding relationships come from the above two modules, which are the effective feature results obtained from the FL feature analysis. The triples of malicious behavior feature graph are shown in Table 4, including 4 entities, 1 DDoS attack, 5 sub-attacks, 22 attack types and 79 flow features. The knowledge graph after FL will increase the corresponding relationship between sub-attacks and flow features, which reflects the effective features of the 5 sub-attacks divided in our work. The knowledge graph will provide feature selection guidance of the five sub-attacks for coarse-grained detection.

#### 3.4.3. Distributed Construction

The distributed construction of knowledge base is based on blockchain. See Section 3.3.3 for the specific transmission process.

### 3.5. Calls between Modules

**Figure 6:**
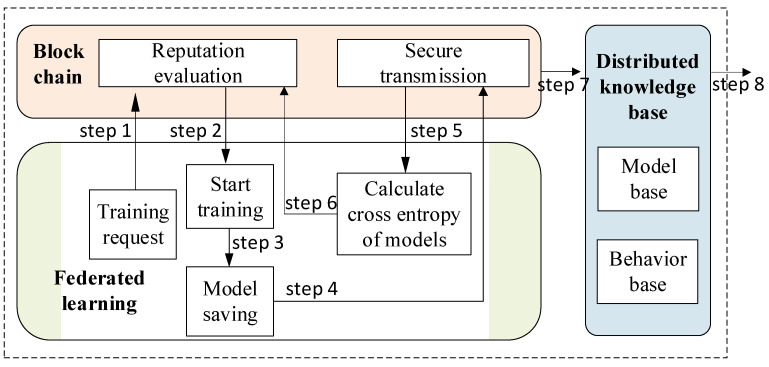
Call framework diagram of module.

In the process of multi-domain DDoS detection based on trusted FL, collaborative implementation of the federated learning module, blockchain module and distributed knowledge base module is required. Therefore, in this section, we mainly explain the call relationship between the above three modules, as shown in Figure 6. In addition, we also give the implementation details of trusted federated learning after merging blockchains, as well as the details of the interaction process with the knowledge base.

We first describe the roles of participants in FL, including guest, arbiter and host. The guest of the task is the task requestor, who controls the training process to a certain extent, including task start, task end and task information uploading to the blockchain. The arbiter of FL can be designated by the guest, and responsible for model aggregation and update. Hosts are the other domains that cooperate in FL.

The steps are as follows:

Step 1. The current domain who applies for an FL task initiates the training request as a guest, and specifies the arbiter and the hosts of the task. Arbiter is responsible for the aggregation of the multi-domain model, and hosts are responsible for the collaborative training of the DDoS detection model.

Step 2. After the job application is uploaded to the blockchain, the blockchain will judge the registration status, online status and reputation evaluation of all participants, and return the corresponding status and reputation value of each participant to ensure that they are safe and credible. Meanwhile, if the reputation evaluation is passed, return the data description owned by all participants, complete the training-related configuration, and start the training task.

Step 3. The FL task includes two main steps: local training and security aggregation. The above steps are trained iteratively until convergence or the reaching of the max iterations. The algorithm of step 1–3 is described as Algorithm 2.
**Algorithm 2** Algorithm of FL joint reputation evaluation**Input:   config file** 1: parse Arguments: partyID of guest, host, arbiter ← config file; 2: **for** i in (guest, host, arbiter) **do**: 3:   if getDevices(i): 4:     if getOnlinestatus(i): 5:       DataMsg = getDataMsg(i); 6:   reputation = calReputation(i); 7:    return DataMsg, reputation; 8:   else: 9:     return Null; 10: **end for** 11: if reputation > default: 12:   run Algorithm 1 with DataMsg and application config file;

Step 4. After training, all participants will save the trained model to the local domain, so as to deploy the trained DDoS detection model to the intrusion detection system.

Step 5. Transmit the final model to guest through the secure transmission mechanism of blockchain for it to calculate the cross entropy of all models.

Step 6. The guest uploads the cross entropy of the model of the last iteration of all participants to the blockchain, then the blockchain audits this job, and the smart contract updates its interactive reputation and the comprehensive reputation of each participant, shown as Algorithm 3.
**Algorithm 3** Job audit and reputation update algorithm**Input:** jobID, guest, host, arbiter, crossEntropy, dataMsg 1: **for** i in (guest, host, arbiter) **do:**
2:   if getDevices(i): 3:     devices[i].frequency++; 4: **end for** 5: FLjob(jobID, guest, host, arbiter, dataMsg); 6: crossEntropy[jobID]= crossEntropy; 7: **for** i in (guest, host, arbiter) **do**: 8:   calcu_rvi(i); 9:   calcu_reputation (i); 10: **end for**

Step 7. Store the trained FL model and effective feature knowledge into the distributed knowledge base based on blockchain.

Step 8. The knowledge base provides guidance for subsequent DDoS detection and assists in the deployment and online detection of the model at the gateway.

## 4. Experiment and Result Analysis

In this section, we implemented the prototype system of trusted FL DDoS detection, using FATE as the main framework of FL. Firstly, we validate the detection methods proposed for 22 attack types. Secondly, we analyze the influence of non-iid datasets on accuracy. At the same time, we simulate the FL attack to test the reputation evaluation method and judge the impact of the threshold on the experimental results. The trusted multi-domain DDoS detection based on FL is carried out under the condition of an optimal threshold and compared with the scheme without reputation evaluation. Then, we compare the difference in detection rate between FL and their corresponding ML algorithms. Finally, we verify the time cost and the update of the knowledge graph of our scheme.

### 4.1. Experimental Environment

**Figure 7:**
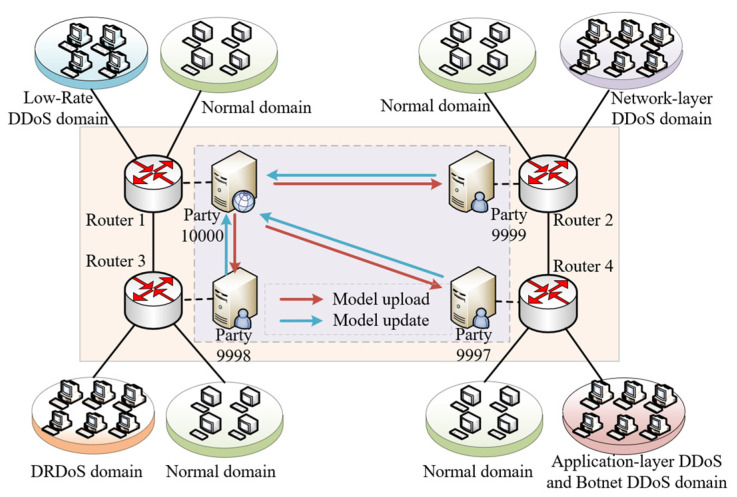
Experimental topology.

In order to verify the detection effect of this method for multi-domain DDoS, a virtual platform based on VMware vSphere is built as the experimental environment. The experiment mainly involves four virtual machines, which are the participants of FL, of which party 10,000 is the aggregator at the same time. The topology of the experiment is shown in the Figure 7, and based on this topology, the deployment of an enterprise-oriented clustered FL environment is realized.

The trusted FL experiment in this paper is based on the framework of FATE (https://github.com/FederatedAI/FATE/ (accessed on 7 October 2022)) and Ethereum (https://www.ethereum.org/ (accessed on 7 October 2022)). The ML experiment is based on TensorFlow (http://tensorflow.google.cn/install/ (accessed on 7 October 2022)), the software environment is ubuntu18.04 operating system, the number of virtual cores is 8, and the memory is 150 GB. It should be noted that the attack domains and its corresponding party host belong to the same domain, and the gateway connected is responsible for collecting data in this domain.

Taking party 10,000 as an example in Figure 7, the dataset of this domain is collected at gateway router 1. The attack type of this domain is mainly low-rate DDoS attacks. Therefore, if data sharing with other domains is not carried out, the detection ability of the trained model for other types will be limited. We use the dataset collected above to test the performance of this paper.

### 4.2. Evaluation Indicators

Precision, recall and accuracy are used to evaluate the model, the calculation formulas are shown as Formulas (12)–(14), in which true positive (*TP*) refers to the number of data pieces that the label belongs to *i* and the predicted result also belongs to *i*. False positive (*FP*) refers to the number of data pieces whose labels do not belong to *i* prediction results belong to *i*. True negative (*TN*) refers to the number of data pieces whose labels belong to *i* and the predicted results do not belong to *i*. False negative (*FN*) refers to the number of data pieces whose labels do not belong to *i* and the predicted results neither belong to *i*.

(12) $$Precision=\frac{TP}{TP+FP}$$

(13) $$Recall=\frac{TP}{TP+FN}$$

(14) $$Accuracy=\frac{TP+TN}{TP+FP+TN+FN}$$

### 4.3. Performance Analysis

#### 4.3.1. Comparison of Multi-Domain DDoS Detection Algorithms

In order to describe the performance of the model in multi-domain DDoS detection, we design multi classification detection experiment, test the simulated five attack categories and normal flow including 22 attack types, and compare the HomoCNN method used in this paper with HomoSecureBoost [37] and HomoDNN. We set the parameters in Table 5.

**Figure 8:**
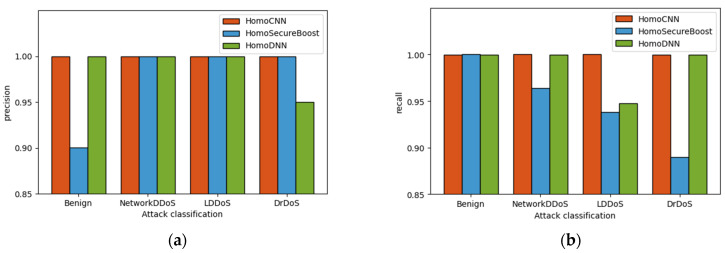
Comparison of different methods: (**a**) Comparison of precision; (**b**) Comparison of recall.

Figure 8 shows the precision and recall rate of the participants who perform best in the multi classification experiment using the three algorithms mentioned above. We can observe that the HomoCNN model is better than the other two models in terms of precision and recall rate. For normal traffic, the HomoCNN model used in this paper has obvious advantages over the Homo secure boost model. For network layer DDoS, LDDoS and Botnet DDoS attacks, the performance of the three algorithms is relatively excellent. For DRDoS attacks, our algorithm has higher precision than HomoSecureboost and higher recall than HomoDNN. In general, the HomoCNN algorithm used in this paper can achieve a precision rate of more than 95% for four types of attacks and normal traffic, and can reach 80% precision for DRDoS.

#### 4.3.2. Comparison of Dependent Identically Distributed Data

We test the performance of datasets with different label proportions to verify how non-iid data affect experimental results. We add a small amount of information of other attack domains into the local dataset required for the experiment as seed data D to test the relationship between accuracy and the quantized value γ = D/G × 100%, where the number of the local dataset is recorded as G, the value γ reflects the ratio between the number of the label who has the lowest proportion and the label who has the highest proportion. Table 6 shows the dataset designed at γ = 1%, the label and number of training and prediction datasets used by each training party are shown in the Table 6.

**Figure 9:**
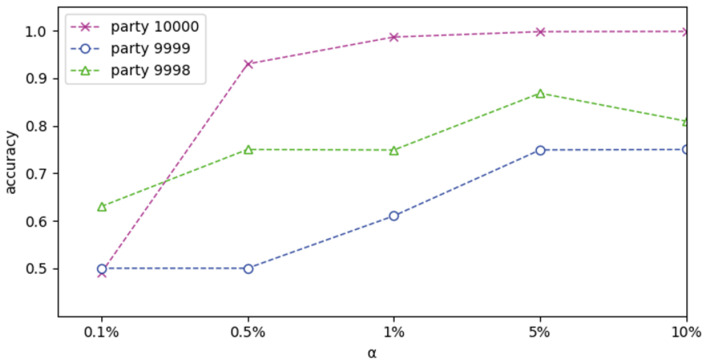
Growth trend of accuracy with γ.

Increase the value γ, make γ from 0.1% to 10%, then we use three participants for training. The accuracy of the three parties is shown in Figure 9. We can see that the accuracy of the three participants all increases in turn with the increase of γ, and it tends to be stable when γ is between 1% and 5%. At this time, it can achieve high accuracy, especially in party 10000.

#### 4.3.3. Comparison of Poisoning Intensity of Malicious Participants

We simulate the data poisoning attack, using the ratio of data poisoning to represent the intensity of the attack, and test the impact of poisoning intensity on FL. We used the HomeCNN method in Section 4.3.1 to detect DDoS attacks with four participants, and the parameter settings are the same as the Section 4.3.1. In order to show the impact of poisoning attack on the participants, we verified the accuracy of the worst performing party.

**Figure 10:**
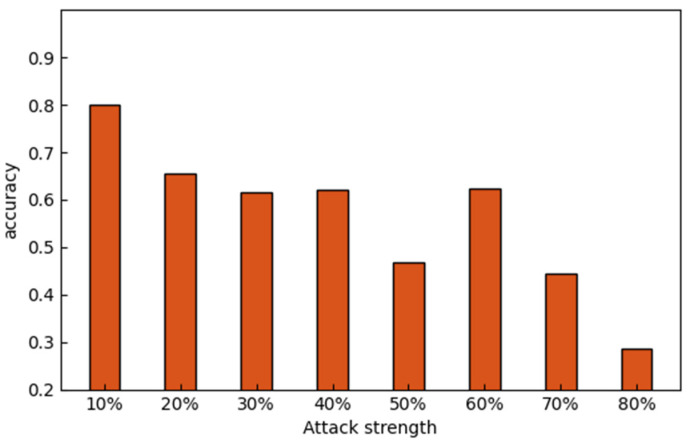
Influence of data poisoning attack strength on accuracy.

As shown in Figure 10, the accuracy of the worst party tends to decrease with the increase of data poisoning intensity, which means that the accuracy rate of participants will decrease with the increase of data poisoning intensity. If untrusted participants launch a data poisoning attack, the accuracy of learning will be negatively affected to a large extent.

#### 4.3.4. Comparison of Reputation Evaluation Methods

Based on the experiment of the data poisoning attack, we verify the effectiveness of the reputation evaluation method proposed in our paper. After the completion of every FL task, we evaluate the reputation of the participants. In the beginning, we set the parameters for calculating reputation in the experiment in Table 7, and the specific meanings of these parameters can be found in Section 3.3.2.

**Figure 11:**
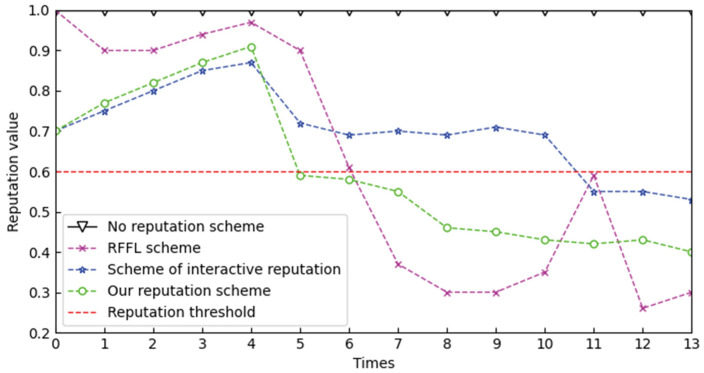
Scheme comparison of reputation value change.

Figure 11 shows the changes in the reputation value of malicious participants before and after the poisoning attack. We add a data poisoning attack during the training time of five and use the following schemes to compare with the scheme in this paper: the scheme without reputation evaluation, the RFFL Scheme [34] and the scheme of interactive reputation.

The scheme without reputation evaluation: the reputation value of the participants remains unchanged at 1.0, which does not have the ability to identify the malicious participants.

RFFL scheme: The cosine similarity between the local model and the global model is taken as the reputation value.

The scheme of interaction reputation: Only considers the cross entropy of the interaction model and does not consider the data reputation and resource reputation.

The results show that in the fifth experiment, that is, when the poisoning attack is just added, the reputation value of the scheme without reputation evaluation will not change. In the fifth experiment, although the reputation of the RFFL scheme is reduced, it is still above 0.8. In the sixth experiment, the reputation value is reduced significantly, but still above 0.6. At the same time, in the eleventh experiment, the reputation value is unstable. The reason is that when the number of trusted participants participating in the training is not enough, the global model cannot be a good representative of the trusted model, therefore, the cosine similarity between the global model and the untrusted model is not enough to reflect the difference between the trusted participant’s model and the untrusted model stably. Compared with the scheme in this paper, the scheme of interaction reputation cannot detect the data poisoning attack effectively so the change slope of the reputation value is relatively small, for the reason that it does not consider the data reputation, and the dataset of the attacker in the data poisoning attack is often of poor quality.

To sum up, we set the reputation judgment threshold β to 0.6, See Section 3.3.2 for details of β. The experimental results show that our scheme can better judge poisoning behavior than the other two schemes when adding poisoning attack, so as to reduce its reputation value to less than 0.6 and can reduce its reputation value every time it launches a poisoning attack steadily. In addition, based on blockchain, our scheme makes the reputation value more transparent.

**Figure 12:**
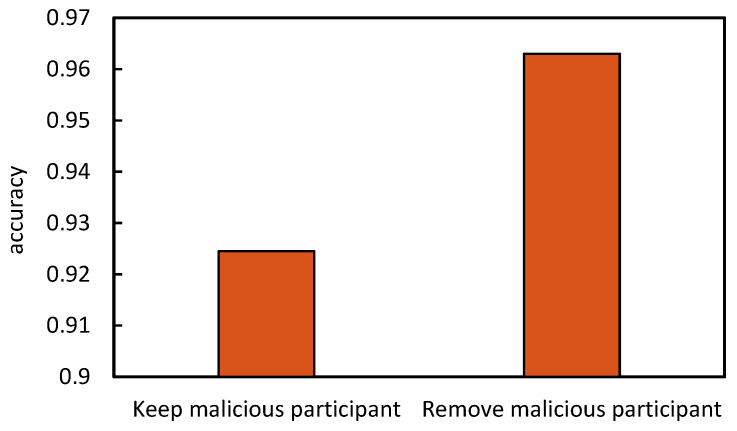
Comparison before and after removing the malicious participant.

Finally, we eliminate the participant whose reputation is less than β, as shown in Figure 12, the experimental results show that the accuracy of the best performing party is improved by 3%–4% compared with that before the malicious party is eliminated.

#### 4.3.5. Performance Comparison between FL and ML

In order to compare the performance of the FL scheme proposed in this paper, we use the corresponding ML CNN to make a comparison. We design two experiments: uploading the datasets of each domain to a central party for centralized training and using local datasets for training in three participants, respectively.

**Figure 13:**
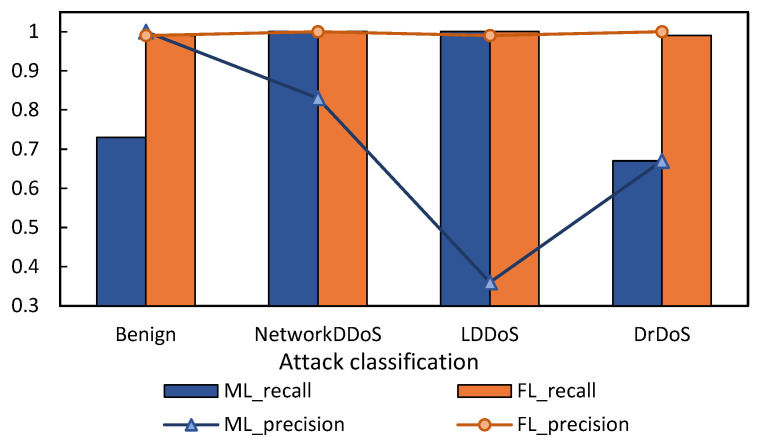
Comparison of FL and local ML without uploading datasets.

We use the average of the accuracy and recall of the three local models trained to reflect the comprehensive effect of local detection. Figure 13 shows the comparison between the FL scheme proposed in this paper and the local ML training without dataset sharing. When the local domain dataset is used simply, ML can only detect the category of attacks that have occurred and normal traffic locally. As shown in the figure, since LDDoS attacks do not occur in the other two domains, the average accuracy of LDDoS in the three domains is not high, but the normal traffic can be effectively detected for they occur in all three domains. This figure reflects that the FL scheme proposed in this paper can effectively detect the attack categories that do not appear in the domain.

**Figure 14:**
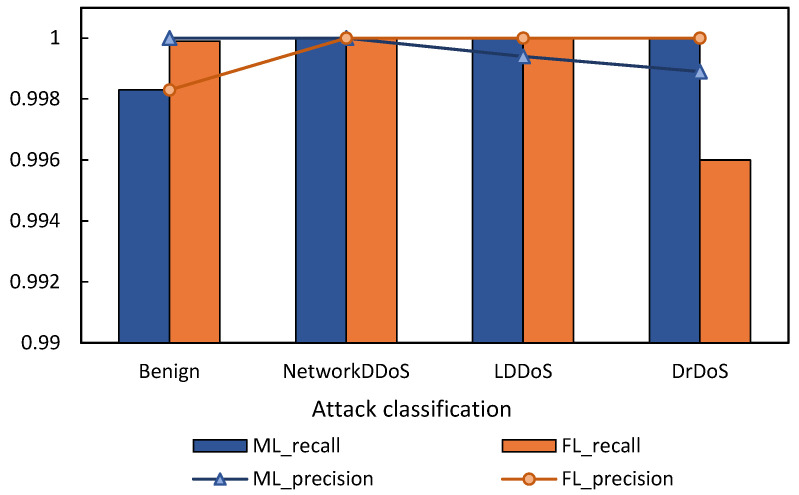
Comparison of FL and ML with uploading datasets.

Figure 14 shows the comparison between the HomoCNN model of FL and the CNN model of ML. As we can see, our FL method can achieve the same effect as the ML model trained by concentrating data on one client, but it does not need to contribute all the data. Therefore, FL can provide an effective detection scheme for multi-domain DDoS Federation.

#### 4.3.6. Time Cost Analysis

**Figure 15:**
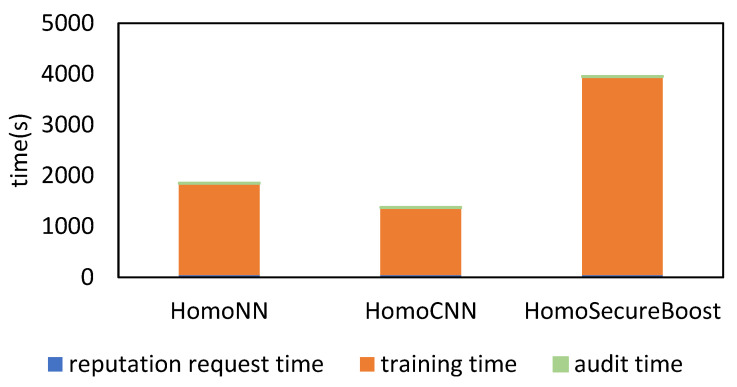
Comparison of the time cost of different models.

In order to verify the time cost of our scheme after joining the blockchain, we analyzed the time required for our scheme, include the time of repudiation request, training and audit. The experimental results are shown in Figure 15. The repudiation request time refers to the connection time, request data and request reputation time of Ethereum. The training time is the training duration of the three models, HomoNN, HomoCNN and HomoSecureBoost, with the same parameters as Section 4.3.1. The audit time includes the broadcast time of the transaction, the queue time of transaction and the block out time. The broadcast of transactions is mainly determined by the number of nodes built in the network and the point-to-point communication delay between network nodes, and the queuing and block out time are mainly determined by the consensus algorithm of the blockchain system and its parameters which are configured according to the mining difficulty and block size when the blockchain is initialized. To sum up, since we consider the time delay caused by multiple interactions in other schemes, our scheme only considers one interaction per training, hence the training time is the most critical time in the whole. Therefore, we believe that the advantages of blockchain to this scheme are obviously higher than the impact of its delay.

**Figure 16:**
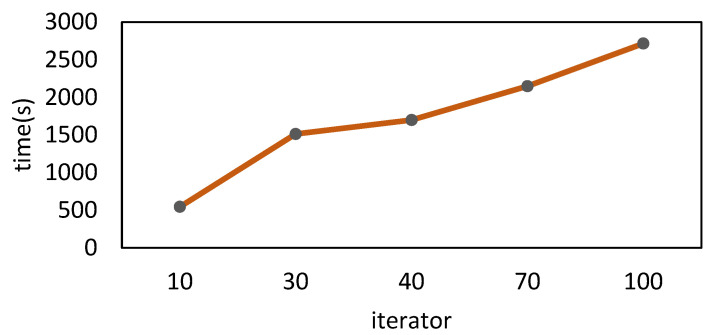
Comparison of different iterators on HomoCNN training time.

Finally, we analyze the training duration of the HomoCNN algorithm in different iterations, as shown in Figure 16. It can be seen that the training time is almost proportional to the number of iterations, because each additional iteration requires an additional communication between the aggregator and all other participants.

#### 4.3.7. Comparison before and after Knowledge Base Update

**Figure 17:**
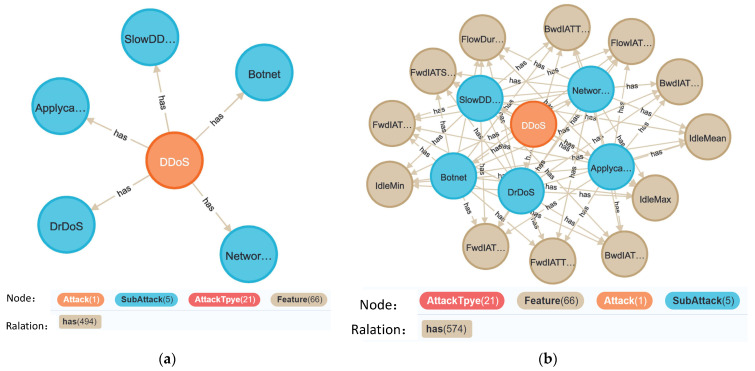
The update of malicious behavior feature graph: (**a**) Behavior malicious feature graph before update; (**b**) Behavior malicious feature graph after update.

The comparison diagram before and after the update of the malicious behavior feature graph of the distributed knowledge base is shown in Figure 17. Before the update, the malicious behavior feature graph includes the relationship between attack type and feature, with a total of 494 relationships. After FL training, the knowledge base will be updated, and the updated knowledge graph adds 80 relationships, which is the connection relationship between sub-attack and feature.

## 5. Conclusions

In this paper, we focus on the reliable application of FL to multi-domain DDoS detection. We first introduce a multi-domain DDoS detection method, which realizes the detection of 22 types of DDoS attacks and classifies them into 5 categories, with most of them reaching more than 95%. To ensure the reputation of FL participants, we introduce blockchain to evaluate the reputation of participants from three aspects: interaction reputation, data reputation and resource reputation, which has a higher ability to identify malicious participants against the data poisoning attack when the threshold is set to 0.6. In addition, by comparing with ML, our scheme has a higher precision and recall rate than the local detection scheme without dataset transmission and pays more attention to dataset protection than the scheme that transmits datasets to the central training party, with the similar accuracy and recall rate. In addition, most of the time spent on multi-domain DDoS detection combined with blockchain is spent on the process of FL, and the time spent interacting with blockchain is greatly reduced because we only evaluate the model of last iteration. Finally, we combine the model trained using our method with the knowledge base to build and update the feature graph of behavior, so as to save the global knowledge and provide knowledge support for detection.

In the future, in order to further improve the detection accuracy, we can further study how to mitigate the impact of non-iid datasets. At the same time, more weight parameters can be considered to optimize the reputation evaluation process and achieve more reliable FL detection.

## Figures and Tables

**Table 1:** Existing Survey on DDoS detection with ML/FL and our new contribution.

Areas	Paper	DDoS Detection with ML/FL						Highlights
		Data Set Type	Real-Time Detection	Categories Diversity	Supervised/ Unsupervised	Poisoning Attack	Solve Non-iid Problem	
ML	[14]	Flow	N	N	supervised	Not involved	Not involved	A method to detect flooding attacks
	[18]	Flow/package	N	Y	supervised	Not involved	Not involved	Propose a more comprehensive DDoS detection classification
	[5,6,7,8,9]	Flow	Y	N	supervised	Not involved	Not involved	Detect specific types of DDoS such as network layer and application layer respectively. An online detection method based on time window is proposed
	[19]	Flow	Y	N	Semi-supervised	Not involved	Not involved	Present an online sequential semi-supervised ML approach for DDoS detection
	[20]	Flow	N	N	unsupervised	Not involved	Not involved	Propose a hybrid unsupervised DL approach using the stack autoencoder and One-class Support Vector Machine for DDoS attack detection.
	[22]	Flow	Y	N	supervised	Not involved	Not involved	Provide a real-time DDoS detection scheme in SIP system
FL	[26]	Flow	N	N	supervised	N	N	Propose an effective intrusion detection method based on FL-LSTM framework
	[23]	Flow	N	N	supervised	Y	N	Describe a method of licensing joint learning based on blockchain Put forward the method of auditing machine learning model
	Our work	Flow	Y	Y	supervised	Y	Y	Design datasets for multi domain learning and implement FL DDoS training Propose reputation evaluation scheme based on blockchain

**Table 2:** Attack datasets.

Attack Type	Quantity	Sub-Attack (Label)	Tool
Benign	880,693	Benign	socket package (https://docs.python.org/3/library/socket.html (accessed on 15 April 2021))
ACK	47,535	Network layer DDoS	hping tool (https://github.com/antirez/hping (accessed on 7 October 2022))
UDP	47,066		
SYN	45,450		
SlowBody	110,043	LDDoS	Slow HTTPTest (https://github.com/shekyan/slowhttptest/(accessed on 7 October 2022))
SlowRead	45,389		
SlowHeaders	100,793		
Shrew	68,074		Scapy (https://scapy.net (accessed on 7 October 2022))
CC	253,524	Application layer DDoS	Webbenchbench (http://www.ha97.com/4623.html (accessed on 7 October 2022))
HTTP-Flood	63,435		Golden-Eye (https://github.com/jseidl/GoldenEye/(accessed on 7 October 2022))
HTTP-Post	56,887		
HTTP-Get	51,556		
TFTP	42,059	DRDoS	Scapy
Memcached	39,021		
DRDoS_SSDP	22,035		
DRDoS_NTP	9430		
Chargen	23,077		
DRDoS_SNMP	15,576		
Ares	709,748	Botnet DDoS	Ares (https://github.com/sweetsoftware/Ares/(accessed on 7 October 2022))
BYOB	2926		BYOB (https://github.com/malwaredllc/byob/(accessed on 7 October 2022))
Miral	2251		Miral (https://github.com/jgamblin/Mirai-Source-Code (accessed on 7 October 2022))
Zeus	273		Zeus (https://github.com/Visgean/Zeus (accessed on 7 October 2022))
IRC-Botnet	42		https://github.com/jpdias/botnet-lab (accessed on 7 October 2022)

**Table 3:** Attributes of information management.

Attribute	Variable	Describe
Participants identity attributes	owner_ethaddr	Ethereum address of participant
	timestamp	Timestamp that device created
	latest_timestamp	Timestamp the latest online
	party_id	Participant id
	domain_name	Participant name description
	online	Online status
	data_quantity	Data quantity stored in each device
	frequency	Training times
Data attributes	owner_ethaddr	Ethereum address of dataset owner
	owner_partyid	Party_id of dataset owner
	namespace	Data description: namespace
	table_name	Data description: table_name
	data_entropy	Information entropy of dataset
	data_quantity	Quantity of dataset
Job attributes	job_id	ID of job
	guest	Party_id of guest
	arbiter	Party_id of aggregator
	host	Party_id of host
	job_data	Data used in training
	cross_entropy	Cross entropy of all models

**Table 4:** Triples of malicious behavior feature graph.

Entity	Attribute	Relationship
DDoS attack	DDoS attack description	Include sub-attack
Sub-attack	Description of five categories	Associate attack type and flow feature
Attack type	Description of 22 attack types	Associate flow feature
Flow feature	Description of 79 flow features	Correspond sub-attack and attack type

**Table 5:** Parameter setting.

Parameter	HomoCNN	Homosecure Boost	HomoDNN
learning_rate	0.01	0.04	0.01
max_iter	100	-	55
optimizer	Adam	-	RMSprop
aggregate_every_n_epoch	1	1	1
subsample_feature_rate	-	0.9	-
num_trees	-	3	-
max_depth	-	7	-

**Table 6:** Federated learning dataset of all parties at γ = 1%.

Party Id	Category (Label)	Training Datasets	Prediction Datasets
9999	Benign	50,000	30,000
	NetworkDDoS	50,000	30,000
	LDDoS	499	30,000
	DRDoS	499	30,000
10000	Benign	50,000	30,000
	NetworkDDoS	499	30,000
	LDDoS	50,000	30,000
	DRDoS	499	30,000
9998	Benign	50,000	30,000
	NetworkDDoS	499	30,000
	LDDoS	499	30,000
	DRDoS	50,000	30,000

**Table 7:** Parameters for calculating reputation.

Parameter	Value	Parameter	Value
*b*_1_	100	*a*_1_	0.5
*k*_1_	2.898	*a*_2_	0.3
*b*_2_	40	*a*_3_	0.2
*k*_2_	600	-	-
